# Biological Properties of Bee Bread Collected from Apiaries Located across Greece

**DOI:** 10.3390/antibiotics10050555

**Published:** 2021-05-10

**Authors:** Nikos Asoutis Didaras, Ioannis Kafantaris, Tilemachos G. Dimitriou, Chrysanthi Mitsagga, Katerina Karatasou, Ioannis Giavasis, Dimitris Stagos, Grigoris D. Amoutzias, Fani Hatjina, Dimitris Mossialos

**Affiliations:** 1 Laboratory of Microbial Biotechnology, Molecular Bacteriology, Virology, Department of Biochemistry & Biotechnology, School of Health Sciences, University of Thessaly, 41500 Larissa, Greece; didasout@uth.gr (N.A.D.); kafantarisioannis@gmail.com (I.K.); tidimitr@bio.uth.gr (T.G.D.); 2 Laboratory of Food Microbiology and Biotechnology, Department of Food Science and Nutrition, University of Thessaly, 43100 Karditsa, Greece; cmitsagga@uth.gr (C.M.); igiavasis@uth.gr (I.G.); 3 Apicultural Centre of Larissa, Federation of Greek Beekeepers Associations, 41222 Larissa, Greece; omse@otenet.gr; 4 Laboratory of Animal Physiology, Department of Biochemistry & Biotechnology, School of Health Sciences, University of Thessaly, 41500 Larissa, Greece; stagkos@bio.uth.gr; 5 Bioinformatics Laboratory, Department of Biochemistry and Biotechnology, School of Health Sciences, University of Thessaly, 41500 Larissa, Greece; amoutzias@bio.uth.gr; 6 Department of Apiculture, Institute of Animal Science, Hellenic Agricultural Organisation DEMETER, 63200 Nea Moudania, Greece; fhatjina@instmelissocomias.gr

**Keywords:** bee bread, bee product, antioxidant, antibacterial, functional food

## Abstract

Bee bread is the only fermented product of the beehive. It constitutes the main source of proteins, lipids, vitamins, and macro- and microelements in honeybee nutrition and it exerts antioxidant and antimicrobial properties, though research on these aspects has been limited so far. In this study 18 samples of Greek bee bread, two of which were monofloral, were collected during different seasons from diverse locations such as Crete and Mount Athos and were tested for their bioactivity. Samples were analyzed for their antibacterial properties, antioxidant activity, total phenolic content (TPC), and total flavonoid content (TFC). The antimicrobial activity of each sample was tested against *Staphylococcus aureus*, *Pseudomonas aeruginosa*, *Klebsiella pneumoniae*, and *Salmonella typhimurium*. Our data demonstrate that all samples exert inhibitory and most of them bactericidal activity against at least two pathogens. Furthermore, all samples exert significant antioxidant activity, where the monofloral *Castanea Sativa* sample demonstrated superior antioxidant activity. Nevertheless, the antioxidant and antimicrobial activity were not strongly correlated. Furthermore, machine learning methods demonstrated that the palynological composition of the samples is a good predictor of their TPC and ABTS activity. This is the first study that focuses on the biological properties of Greek bee bread and demonstrates that bee bread can be considered a functional food and a possible source of novel antimicrobial compounds.

## 1. Introduction

Honeybee products (honey, bee-collected pollen, bee bread, royal jelly, beeswax, and bee venom) have been used as folk remedies since ancient times. Nowadays, depending on national legislation, they are considered foods, food supplements, superfoods, functional foods, or even complementary medicines [1,2,3].

Honey is definitely the most studied bee product regarding its antimicrobial properties. It is reported to exert antibacterial [4,5,6,7], antifungal [8], and antiviral activity [9,10,11]. In addition, it exhibits anti-inflammatory and wound-healing action [12], as well as anticancer properties [13,14,15]. Its antioxidant properties vary in tandem with its botanical origin [16,17,18]. Furthermore, honey is considered a prebiotic food positively affecting the human microbiome and well-being [19,20,21].

Bee-collected pollen (BCP) is well known for its high nutritional value, as it provides carbohydrates, proteins, essential amino acids, fatty acids (including ω-3 and ω-6 fatty acids), vitamins, and macro- and microelements. Furthermore, BCP contains a plethora of bioactive compounds such as polyphenols, triterpenes, carotenoids, phospholipids, phytosterols, bioactive peptides, organic acids, prebiotics, and probiotics [22,23,24]. Some of these compounds are secondary metabolites protecting plant male gametes, inside the pollen grains, from herbivores, pollen thieves, pathogens, and heat stress [25,26,27]. Others are derived from bee secretions and added microbiota [1]. This wealth of nutrients and health-promoting compounds (and possibly their synergistic action) is responsible for the antioxidant, hepatoprotective, cardioprotective, anti-inflammatory, anti-carcinogenic and antimicrobial properties attributed to BCP [28,29,30,31].

Nevertheless, the bioavailability of these compounds is not easily determined, as they are enclosed inside the walls of pollen grains, which are difficult to digest. Exine, the outermost wall of pollen grains, is made of sporopollenin, a resistant biopolymer, whereas intine, the inner layer, is made of cellulose microfibrils and pectin [32]. Therefore, in order to increase the digestibility of pollen and the bioavailability of its nutrients and bioactive molecules, bench fermentation of pollen has been attempted and several food products have been developed [33,34,35,36,37].

Bees have been fermenting pollen for millions of years, producing bee bread (BB) probably out of necessity to preserve pollen of high quality, which is not available throughout the year, and to increase the bioavailability of pollen nutrients. Pollen is of paramount importance to the survival of the bee colony, as it constitutes the only source of protein and lipids, and the main source of macro- and microelements [38]. Interestingly, bees prefer to feed on BB rather than freshly collected pollen.

Bees make bee bread out of pollen by adding glandular secretions to flower pollen along with some nectar from their crop (honey stomach), and at the same time they inoculate this mixture with their own microbiota [39,40]. Inside the hive, bee-collected pollen is stored in the comb cells, where it soon undergoes solid-state fermentation by lactic acid bacteria and yeasts [40,41,42]. As lactic acid is produced and pH drops, microbial succession occurs and a slower process begins: the maturation of bee bread. During maturation, the predigestion of pollen grains by added bee enzymes as well as enzymes produced by the bee-bread microbiome increase its nutritional value [40]. In this context, it is tempting to assume that the diverse bee-bread microbiota, which also serves as bee food, produce not only enzymes but also amino acids, vitamins, and antimicrobial compounds, thus enhancing the bioactivity of bee bread [43,44,45,46].

In order to elucidate how chemical composition differences might affect nutrient content and bioavailability, BCP and BB samples collected simultaneously from the same hive should be analyzed for comparison. In that regard, Anđelković et al. [47] showed that the content of crude protein was increased by 19.91% in BB compared to BCP. Using a different approach, Kaskonie et al. [43] reported that the total phenolic and flavonoid content as well as the radical scavenging activity increased by 1.27–2.40-fold in BCP, following fermentation.

Taking into account different published studies, BB is at least as nutritious and bioactive as BCP. It is regarded as a functional food with health benefits and therapeutic applications similar to those of pollen [2,3,48]. According to Kieliszek et al. [48], BB is more potent than pollen and therefore is usually administered in smaller doses or for a shorter period of time. Nevertheless, in health-food markets, BB is significantly less available than pollen, which is much easier to collect from the bee hive. The last few years’ research regarding antimicrobial and antioxidant BB properties has started to attract attention [1,49,50,51,52].

The biological (antimicrobial, antioxidant) and nutritional properties of BCP and BB are directly related to their composition, which in turn is directly related to their botanical origin [53,54]. This is the case for other bee products such as honey or even beeswax [55,56,57,58].

Greek flora is characterized by high biodiversity, as it comprises 5885 species and 2000 subspecies, representing 6760 taxa. Of these, 1061 species, 411 subspecies, and 31 varieties are endemic, a total of 1442 taxa [59]. The aim of this study was to assess for the first time the antibacterial and antioxidant properties of BB samples collected from apiaries located across Greece and attempt to correlate them to botanical origin.

## 2. Results

### 2.1. Palynological Analysis

Palynological analysis was performed in all 18 samples in order to investigate whether there is any correlation between the antioxidant, antimicrobial activity and the botanical origin. Table S1 demonstrates the pollen grain content (%) for each sample. Sample 18 was found to be monofloral (99.8% *Castanea sativa* from Mount Athos). Moreover, sample 13 could be considered monofloral (*Cistus* spp. 78%). Clearly dominant plant species/genus were identified in sample 5 (*Hedera helix* 52.,4% and sample 11 (*Borago* spp. 54.8%). Sample 8 consisted of pollen grains belonging mainly to the Brassicaceae family.

### 2.2. Antioxidant Activity

Table 1 presents the IC_50_ values of DPPH and ABTS^•+^ radical scavenging assays employed to assess the antioxidant activity of BB samples. The lower the IC_50_ value, the higher the antioxidant capacity. Regarding the DPPH assay, the IC_50_ values ranged from 0.18 (sample 18) to 1.8 mg/mL (sample 2). Besides sample 18, strong DPPH radical scavenging activity was observed for BB samples 9, 6, 7, and 3. Furthermore, BB samples 4, 11, 16, and 17 demonstrated similar DPPH radical scavenging activity. In contrast, relatively weak DPPH radical scavenging activity, besides sample 2, was observed for BB samples 5 and 1. In the ABTS^•+^ assay, the IC_50_ values ranged from 0.38 (sample 18) to 1.80 mg/mL (sample 2). Apart from BB sample 18, a strong ABTS^•+^ radical scavenging activity was also exhibited by BB samples 9, 6, 10, and 11. In contrast, weak ABTS^•+^ radical scavenging activity was exhibited by BB samples 2, 5, and 13.

### 2.3. Total Phenolic (TPC) and Total Flavonoid Content (TFC)

In Table 1, the total phenolic content (TPC) and the total flavonoid content (TFC) are presented. The TPC of BB samples, as measured by the Folin–Ciocalteu method, ranged from 6.49 (sample 12) to 14.64 mg (GAEs)/g sample (sample 18). BB samples (3, 7, 11, and 17) also exhibited high TPC (Table 1). The TFC of the tested samples, as measured by the aluminum chloride colorimetric method, ranged from 2.56 (sample 10) to 5.49 mg (QE)/g sample.

### 2.4. Antimicrobial Activity

Eighteen BB samples were tested against clinical and food–borne pathogens. All BB samples exerted bacteriostatic activity against all tested pathogens. Moreover, all samples exerted bactericidal activity, except sample 1, against *Salmonella typhimurium*, and samples 1, 6, 16, 17, and 18 against *Klebsiella pneumoniae* (Table 2).

The MIC and MBC values of each sample were identical in 14 out of 18 samples tested against methicillin-resistant *S. aureus* (MRSA), in 16 out of 18 tested against *P. aeruginosa*, in 10 out of 18 tested against *S. typhimurium*, and in 10 out of 18 tested against *K. pneumoniae*. These findings suggest that the antibacterial activity of bee bread is probably due to compounds that cause irreversible damage to bacterial cells. However, the existence of bacteriostatic substances cannot be ruled out.

Eleven out of 18 samples exhibited lower MIC values against *S. aureus* compared to Gram(–) bacteria. In accordance with this observation, the lowest MIC value was recorded against *S. aureus* (sample 14). Of all samples, sample 14 demonstrated the lowest MIC and MBC values against *S. aureus* (3.9 mg/mL), *P. aeruginosa* (15.6 mg/mL), and *S. typhimurium* (7.8 mg/mL). Sample 4 showed the lowest MIC and MBC values (9.9 mg/mL) against *K. pneumoniae* compared to the other samples.

Interestingly, there are samples that exhibited lower MIC values against Gram(–) bacteria compared to Gram(+). For instance, sample 3 showed higher MIC and MBC values against *S. aureus* (48 mg/mL in both cases) compared to the corresponding values against other bacteria (24 mg/mL against *P. aeruginosa*, and 12 mg/mL and 24 mg/mL against *S. typhimurium* and *K. pneumonia*, respectively). Interestingly, sample 5 showed identical MIC and MBC values against all pathogens (23.5 mg/mL).

### 2.5. Statistical Analysis

Statistical analysis was performed aiming to correlate TPC, TFC, and values obtained by DPPH and ABTS^•+^ assays. The analysis determined a significant strong correlation (r = 0.719; *p* < 0.01) between DPPH and ABTS^•+^ radical scavenging assays (Table 3). The analysis also revealed that there was a moderate though significant correlation among the TPC and TFC values (r = 0.583; *p* < 0.05) and a moderate though significant negative correlation between the TPC and DPPH or ABTS^•+^ assays (r = −0.586 and r = −0.512, respectively; *p* < 0.05) (Table 3). Although a significant correlation between the TFC values and free radical scavenging assays was not revealed (Table 3), when looking at TFC and DPPH or ABTS^•+^ values of each BB sample separately, it was observed that some BB samples (e.g., 3, 4, and 11) with high TFC demonstrated high antioxidant activity as well (Table 1).

Similarly, statistical analysis was performed in order to assess a possible correlation between TPC, TFC, DPPH, and ABTS^•+^ radical scavenging activity as well as MIC and MBC values against the four tested pathogens. The analysis revealed no significant correlation between the above-mentioned values regarding all samples and tested microorganisms. However, a weak positive correlation was revealed between the phenolic or flavonoid content and the MIC and MBC values for *S. aureus* and *P. aeruginosa* (Table 4).

Finally, statistical analysis was performed in order to assess the correlation among the TPC, TFC, free radical scavenging, and antimicrobial values and the major pollen families present in each BB sample (Table S1). Only the dominant (>10%) pollen families were included. Moreover, pollen families that were present in less than three samples were excluded from further analysis.

Correlation analysis revealed a significant strong correlation (r = 1.0; *p* < 0.01) between TPC and BB content of the Fagaceae pollen family. Regarding TFC values, no significant positive correlation was observed, though a significant negative correlation (r = −1.0; *p* < 0.01) was revealed between TFC values and Ericaceae pollen content in BB samples. Regarding the free radical scavenging ability, a strong correlation was revealed between Ericaceae and Fagaceae pollen content and DPPH values (r = −1.0; *p* < 0.01) as well as between Rosaceae pollen content and ABTS^•+^ values (r = −1.0; *p* < 0.01).

Regarding the antimicrobial activity of BB samples, a negative strong correlation between *S. aureus* MIC and MBC values and Brassicaceae pollen content was observed (r = 0.90; *p* = 0.01). Ericaceae pollen content was negatively correlated (r = −1.0; *p* < 0.01) with *P. aeruginosa* MIC and MBC values. Moreover, Fabaceae pollen content correlated negatively (r = 0.893; *p* < 0.01) with *P***.** *aeruginosa* MIC values. Finally, t strong negative correlation (r = −0.857; *p* < 0.05) was observed between Fabaceae pollen content and *S. typhimurium* MIC values (Table 5).

### 2.6. Machine Learning Analysis

**Figure 1:**
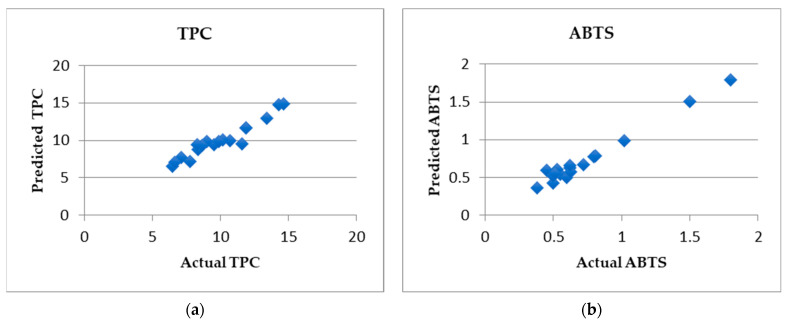
Predicted (based on linear regression) vs. actual values of TPC (**a**) and ABTS (**b**) activity of the samples.

Firstly, we assessed whether the palynological composition could be used to predict any of the antioxidant properties of the samples. Towards this goal, feature selection and linear regression were implemented for all palynological features against each of the four antioxidant-related features (TPC, TFC, DPPH, ABTS) separately. After applying our stringent criteria (see Materials and Methods, Section 4.11), we observed that palynological features could be used to predict the TPC and ABTS values of the samples with R values of 0.85 and 0.89, respectively, based on five-fold cross-validation. The prediction of the linear regression models is depicted in Figure 1. Of note, random forest models also achieved very good performance for the same feature subsets that were used for linear regression for TPC and ABTS (R values of 0.75 and 0.8, respectively). Thus, although the number of samples used in our analyses is considered relatively small for such machine learning analyses, still, there seems to be enough information in the palynological composition to allow for relatively accurate prediction of TPC and ABTS activity. We consider these results as very promising, but they will need to be further validated in the future by a larger number of samples.

We repeated the machine learning analyses in order to investigate whether we could accurately predict any of the antimicrobial activities (MIC, MBC) of the samples against any of the four bacteria or their average activities by using the palynological composition and/or antioxidant activity features. However, none of the models passed all three of our stringent criteria (see Materials and Methods, Section 4.11). Nevertheless, this may be attributed to the rather small number of samples for such computational analyses and it is possible that a future machine learning analysis with more samples may actually reveal palynological features that can accurately predict certain antimicrobial activities.

## 3. Discussion

Bee bread is the least studied bee product regarding its biological properties and rather unknown to most consumers. Relevant studies are scarce, demonstrating high variability of antimicrobial and antioxidant properties [1]. Nevertheless, previous studies conclude that BB should be considered a functional food [2,48,60,61,62]. The variability of biological properties could be attributed to differences in the botanical and geographical origin of the samples as well as to differences regarding the extraction methods used. Another possible explanation could be that most published studies on BB antimicrobial activity test but few samples (1–5) [1] with the notable exception of the very recent study by Pelka et al. [49] on Polish BB. In order to correlate biological properties with botanical origin, a sufficient number of samples is required to represent both botanical diversity and geographical distribution.

**Figure 2:**
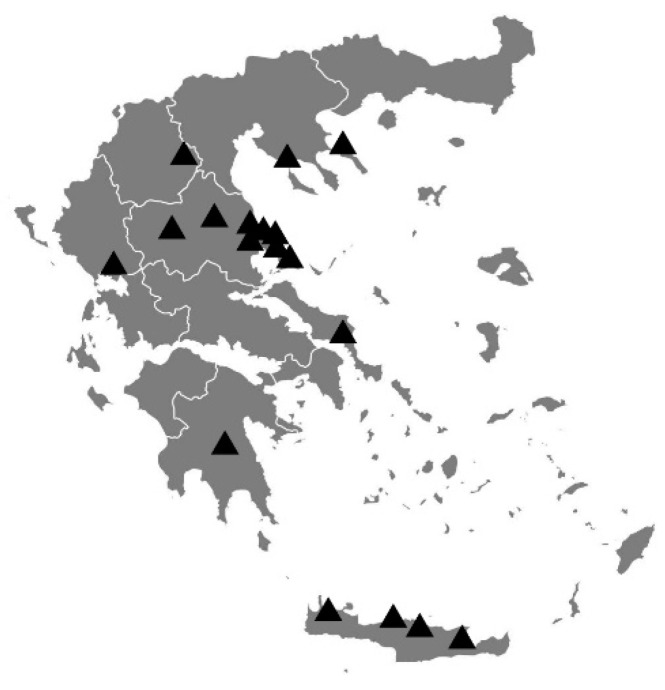
Geographic locations of bee bread samples.

In this study, the first to assess the bioactivity of Greek bee bread, sampling was carefully planned in order to include locations characterized by diverse climate and plant diversity (Figure 2), such as northeastern Crete, where the climate is dry and phryganic ecosystems are dominant, and Kozani and Arta, where the climate is humid and rather cold. Climate differences significantly affect the plant communities from which the bees collect pollen [63,64].

Overall, bee bread has demonstrated high antioxidant activity in previous studies [2,3,48,61,62,65]. Our data demonstrated that Greek bee bread samples exert significant antioxidant activity. Sample 18, a monofloral sample of chestnut bee bread (99.8% *Castanea sativa* from Mount Athos), exhibited the highest antioxidant activity amongst all samples. This is the first time that a monofloral sample of chestnut bee bread was assessed for its biological properties. In DPPH and ABTS^•+^ assays, the IC_50_ values of sample 18 were 0.18 mg/gr and 0.38 mg/gr, respectively. These values rank it among the most powerful antioxidant foods [66]. This is probably associated with the high content of chestnut pollen in phenols, flavonoids, and kynurenic acid, which exert high antioxidant activity [67,68]. Similarly, 16 out of the 18 samples showed IC_50_ values of less than 1 mg/gr in DPPH and/or ABTS^•+^ assays. Samples 2 and 5 showed values close to or slightly above 1 mg/gr in both methods. These results suggest that Greek bee bread could be considered a functional food showing significant antioxidant activity.

It is unknown to what extent the storage conditions of bee bread as well as maturity at the collection time affect its biological properties. Samples 2 and 5 demonstrated the lowest antioxidant activity among the 18 samples. These two samples were stored and collected in a different way than the other samples. Sample 2 was stored for six months before collection, at room temperature inside its comb, which was placed inside an empty hive. In contrast with the other samples, which were collected from frames of mature BB, sample 5 was collected just 11 days after insertion of an empty comb into the hive, presumably long before reaching maturation.

Further research is needed on bee bread post-harvest storage conditions as well as its level of maturity in order to preserve and enhance its biological properties. A recent in vitro study showed that during pollen fermentation under laboratory conditions, there is an increase in phenolic acids, flavonoids, and the antioxidant activity in the fermented product compared to fresh pollen [43]. Therefore, it should be investigated whether this is the case during the conversion of pollen into bee bread in the hive and to what extent the microbiome plays a role in the biological properties of BB. It is plausible that correlation between phenol/flavonoid content and the antibacterial activity of BB was not determined due to different levels of maturity and microbiota contribution in the antimicrobial activity of BB samples. However, other BB compounds than phenols and flavonoids may play a significant role in antimicrobial activity.

Nevertheless, the antioxidant activity of Greek bee bread is statistically correlated to the total content of polyphenols (Table 3). Sample 18, which exhibited the highest antioxidant activity, showed the highest content of polyphenols (Table 1) at the same time.

The antimicrobial activity of Greek bee bread was demonstrated against a food-borne pathogen such as *S. typhimurium*, two important nosocomial pathogens (*P. aeruginosa* and *K. pneumoniae*), and against methicillin-resistant *S. aureus*, which, apart from being a cause of serious infections, is also considered a personal hygiene microbiological indicator in food processing, according to EU legislation (Commission Regulation EC 2073/2005). A heat-resistant enterotoxin is produced by *S. aureus* when it forms colonies, and therefore might often be implicated in food poisoning [69,70]. Moreover, *P. aeruginosa* is also a safety indicator in risk assessment of drinking water, as well as cosmetics and edible pharmaceuticals [71].

All samples demonstrated bacteriostatic activity against all tested pathogens. In most cases the MIC and MBC values were identical, indicating that bee bread exerts mainly bactericidal activity against certain pathogens. BB antimicrobial activity is dose-dependent and variable. In general, *S. aureus* was the most susceptible tested pathogen. However, there were samples, such as sample 3, that showed higher activity against pathogens other than *S. aureus* or demonstrated the same antibacterial activity regardless of the tested pathogen, as was the case of sample 5.

The antimicrobial activity of bee bread was generally attributed to diverse phytochemicals, mainly polyphenols, fatty acids, phytosterols, and presumably microbial metabolites [1]. Pelka et al. [49] reported no correlation between the phenolic content and antimicrobial activity of Polish BB. In accordance, this study does not report a statistically significant correlation between antibacterial and antioxidant activity or the total content of phytochemicals such as polyphenols or flavonoids (Table 4). However, it is plausible that specific phytochemicals contribute to the bacteriostatic or bactericidal activity exerted by BB. This may include microbial metabolites such as organic acids, fatty acids, or polysaccharides present in bee bread, and future research should attempt to identify such compounds.

In addition to phytochemicals, other substances such as proteins or proteinaceous compounds present in BB exert antimicrobial activity (Didaras and Mossialos unpublished data). Identification and quantification of proteinaceous compounds that contribute to BB antimicrobial activity are crucial for understanding BB’s mode of action.

Water suspensions of bee bread samples not subjected to physical or chemical processing was tested in order to assess the antioxidant and antimicrobial properties. These data are useful for the assessment of the bioavailability of BB compounds, considering that humans cannot effectively digest pollen grains. In addition, they might be applied in BB food-safety risk assessment and BB classification as a functional food because they demonstrate antioxidant and antibacterial activity without any additional processing.

Palynological analysis (Table S1) revealed a complex botanical origin of the BB samples tested in this study. Furthermore, the botanical source of BB samples was statistically correlated with exerted bioactivity for the first time (Table 5). A significant correlation was revealed among Ericaceae and Fagaceae pollen content and DPPH values, as well as Rosaceae pollen content and ABTS values. A strong correlation between MIC and MBC values against *S. aureus* and Brassicaceae pollen content was observed (−0.9). Greece accommodates more than 350 species from the Brassicaceae family. This family, also known as the mustard family, includes plants used in traditional medicine such as *Capsella bursa pastoris* [72], *Sinapis nigra*, and *Sinapis alba* [73], along with common but also healthy foods such as cabbage, broccoli, and cauliflower. Brassicaceae and *Sinapis* spp. contain glucosinolates that exert anti-tumor and antimicrobial activity [74,75,76]. They also contain polyphenols, flavonoids, saponines, and triterpens [77]. Ericaceae pollen content significantly correlated with MIC and MBC values against *P. aeruginosa*. In Greece, bees forage mostly on *Erica arborea*, *Erica manipuliflora*, and *Arbutus unendo*. These plants contain tannins, alkaloids, arbutin, carotenes, flavonoids, organic acids, and saponins [78]. Fabaceae pollen content correlates solely with MIC values against *P. aeruginosa*. In addition, significant correlation was observed between Fabaceae pollen content and *S. typhimurium* MIC values. The Fabaceae family includes many bee foraging plants such as *Spartium junceum*, *Robinia pseudacacia*, *Glycyrrhiza glabra*, and *Ceratonia siliqua*.

The identified botanical diversity of BB might also explain the variable antimicrobial activity against specific pathogens exerted by some BB samples. Overall, in-depth study of the chemical composition and the factors that might affect its composition and bioactivity (e.g., botanological or geographical origin, fermentation–maturation process), as well as the mode of action, can lead to diverse BB applications in food preservation, cosmetics, and nutraceuticals.

Conclusively, water suspension of BB exerted significant antioxidant and antimicrobial properties. All samples demonstrated antibacterial activity against all four tested pathogens and our data suggest that BB exerts mainly bactericidal activity. The botanical source of BB samples was statistically correlated with bioactivity for the first time. Botanical diversity of BB might explain the variable antimicrobial activity against specific pathogens. This is the first study to assess the antioxidant and antimicrobial nature of Greek bee bread, which can be considered a functional food and a possible source of bioactive compounds that could be used in food preservation, cosmetics, and nutraceuticals.

## 4. Materials and Methods

### 4.1. Bee Bread Samples

A total of 18 BB samples, harvested from diverse locations in Greece, were provided by individual beekeepers. Seventeen samples were harvested during the 2019 beekeeping period. Sample 2 was collected a year earlier (2018), and the frame was stored in an empty hive, at room temperature, until it was extracted along with the other samples harvested in 2019. Sample 5 was collected from the hive in the fall of 2019, during the flowering of ivy (*Hedera helix*). An empty frame was inserted in a beehive and bee bread was extracted 11 days after its insertion, and fresh pollen was constantly brought in and stored.

Each sample was assigned a unique reference number and details regarding the geographical location, the possible botanical sources, and the date of harvest were also recorded (Table S1 and Figure 2). BB was sampled directly from the honeycombs using plastic tubes equal in diameter to the honeycomb cells and then each sample was removed from the tube using a plunger. All samples were stored in sterile plastic containers at –20˚C. Preliminary data on the botanical source of each BB sample was provided by the beekeepers based on the flora available during the harvest season at the location of the apiary.

### 4.2. Palynological Analysis

Palynological analysis was conducted by CheMa laboratories (Korinthos, Greece) as follows: A quantity of 5–10 mg of each sample was dispersed in 1 mL of deionized water using a vortex mixer. A total of 0.5 mL of each suspension was spread on a 22 × 22 mm area of a microscopy slide and was left to dry at 39 °C. After preheating a cover plate on the hotplate at the same temperature, a drop of glycerol gelatin was transferred to it. This held the pollen in position while the cover glass was lowered onto the dried sediment. The pollen grains were identified using an Euromex BioBlue optical microscope at 400× magnification. For the identification of the pollens, the following databases were used: Pollen Atlas (available at pollenatlas.net), pollen Wiki database, and the pollen library at the CheMa laboratories.

### 4.3. Assessment of the Total Phenolic Content (TPC)

TPC of the BB samples was determined in accordance with a modified protocol of the Folin–Ciocalteu method [79]. Initially, a quantity (0.02 g) of each BB sample was weighed and diluted in 1 mL distilled water. Afterwards, 20 μL of each BB sample was added to a tube containing 1 mL of deionized water. Subsequently, 100 μL of Folin–Ciocalteu reagent were added to the mixture, and the tube was allowed to stand at room temperature for 3 min. Thereafter, 280 μL of 25% *w/v* sodium carbonate solution and 600 μL of deionized water were added to the mixture. Following 1 h of incubation at room temperature in the dark, the absorbance was measured at 765 nm versus a blank containing Folin–Ciocalteu reagent and deionized water only. The optical density of each sample (20 µL) in 25% *w/v* solution of sodium carbonate (280 µL) and distilled water (1.7 mL) at 765 nm was also measured. Measurements were conducted on a Spectro UV-12, MRC, Holon, Israel spectrophotometer. TPC was determined using a standard curve with variable concentrations (25–500 μg/mL) of gallic acid. The results are expressed as mg gallic acid equivalents (GAEs)/g sample using the standard curve (absorbance versus concentration) prepared from authentic gallic acid. The experiments were carried out in triplicates and at least on 2 separate occasions.

### 4.4. Assessment of the Total Flavonoid Content (TFC)

Total flavonoids were determined by using the aluminum chloride colorimetric method, as described by Hassan et al. [80], with minor modifications. Each sample (0.2 g) was extracted with 5 mL of 80% ethanol. The samples were mixed in vortex for approx. 1 min and left overnight at room temperature. After centrifugation at 4000× *g* for 10 min, the supernatant was collected and filtered with plain filter paper and the transparent filtrates (bee bread extracts) were stored in sealed glass tubes for further analysis.

The total flavonoid content was determined using a standard curve of quercetin concentrations (5–100 μg/mL). For the preparation of the standard curve, 100 mg of quercetin were dissolved in 100 mL (1 mg/mL = 1000 μg/mL) of 80% ethanol and then from this stock solution diluted standard solutions were prepared. From each diluted standard solution, 0.5 mL was transferred in glass tubes by adding 1.5 mL of 95% ethanol, 0.1 mL of 10% AlCl_3_, 0.1 mL of 1 M potassium acetate, and 2.8 mL of distilled water. For the blank sample, 0.5 mL of 80% ethanol was transferred in a glass tube with 1.5 mL of 95% ethanol, 0.1 mL of 1 M potassium acetate, and 2.9 mL of distilled water (i.e., 0.1 mL of distilled water was used to replace the 0.1 mL of 10% AlCl_3_). Samples were incubated at room temperature for 30 min before measuring the absorbance of each sample at 415 nm on an MRC Spectro UV-12 instrument. The same procedure was followed for the measurement of bee bread extracts by replacing the 0.5 mL of the standard solutions with 0.5 mL of each sample filtrate after appropriate dilution (1/10 dilution) of the extracts (filtrates) with 80% ethanol. The results are expressed as mg quercetin equivalent (QE)/g dw of extract using the standard curve (absorbance versus concentration) prepared from quercetin. The experiments were carried out in triplicate.

### 4.5. Assessment of Free Radical Scavenging Ability by the Use of the DPPH Radical Assay

The 2,2-diphenyl-1-picrylhydrazyl (DPPH) radical scavenging activity of the bee bread samples was evaluated as previously described [81]. For assessing DDPH, a quantity (0.2 g) of each BB sample was weighed and diluted in 1 mL 99.9% methanol. Briefly, a 1 mL freshly prepared methanolic solution of DPPH radical (100 μM) was mixed with tested bee bread samples at different concentrations following dilution in methanol. The contents were vigorously mixed, incubated at room temperature in the dark for 20 min, and the absorbance was read at 517 nm. Measurements were conducted on an MRC Spectro UV-12 instrument. In each experiment, the tested sample alone in methanol was used as blank and DPPH radical alone in methanol was used as control. The percentage of radical scavenging capacity (RSC) of the tested samples was calculated according to the following equation: RSC (%) = [(A_control_ − A_sample_)/A_control_] × 100, where A_control_ and A_sample_ are the absorbance values of the control and the tested samples, respectively. Moreover, in order to compare the radical scavenging efficiency of the samples, the IC_50_ value showing the concentration that caused 50% scavenging of DPPH radical was calculated from the graph plotted RSC percentage against sample concentration. All experiments were carried out in triplicate and at least on 2 separate occasions.

### 4.6. Assesment of Free Radical Scavenging Ability by the Use of the ABTS^•+^ Radical Cation Assay

The free radical scavenging activity of the bee bread samples was further determined by 2,2′-azino-bis (3-ethylbenzothiazoline-6-sulphonic acid) (ABTS) radical cation (ABTS^•+^) decolorization assay as previously described by Cano et al. [82], with some modifications. For assessing ABTS, a quantity (0.2 g) of each BB sample was weighed and diluted in 1 mL distilled water. Briefly, ABTS^•+^ radical was produced by mixing 2 mM ABTS with 30 μM H_2_O_2_ and 6 μΜ horseradish peroxidase (HRP) enzyme in 50 mM PBS (pH 7.5). Immediately following the addition of the HRP enzyme, the contents were vigorously mixed, incubated at room temperature in the dark, and the reaction was monitored at 730 nm until stable absorbance was obtained. Subsequently, 10 μL of different sample concentrations diluted in distilled water were added in the reaction mixture and the decrease in absorbance at 730 nm was measured. In each experiment, the tested sample alone containing 1 mM ABTS and 30 μM H_2_O_2_ in 50 mM PBS (pH 7.5) was used as a blank, whereas the formed ABTS^•+^ radical solution alone with 10 μL H_2_O was used as a control. The RSC percentage and the IC_50_ values were determined as described above for the DPPH method. All experiments were carried out in triplicate and at least on 2 separate occasions.

### 4.7. Bacterial Strains and Growth Conditions

The antibacterial activity of BB samples was tested against methicillin-resistant *Staphylococcus aureus* strain 1552, carbapenem-resistant *Pseudomonas aeruginosa* strain 1773, *Salmonella typhimurium*, and *Klebsiella pneumonia*. All strains were identified and characterized by standard laboratory methods (kindly provided by Prof. Spyros Pournaras, School of Medicine, National and Kapodistrian University of Athens, Athens, Greece). The bacteria were routinely grown in Mueller-Hinton Broth (Lab M, Bury, UK) or Mueller-Hinton agar (Lab M) at 37 °C.

### 4.8. Determination of Minimum Inhibitory Concentration (MIC)

The minimum inhibitory concentration (MIC) of the BB samples was determined in sterile 96-well polystyrene microtiter plates (Kisker Biotech GmbH & Co. KG, Steinfurt, Germany) using a spectrophotometric bioassay as previously described [7], with some modifications. Briefly, 0.5 g BB sample was suspended in sterile ddH_2_O (2 mL final volume) for one hour at room temperature with occasional vortex and then centrifuged at 10,000× *g* for 7 min. The aqueous phase filtered through a 0.22 μM syringe filter and used for serial dilutions in Mueller-Hinton broth corresponding from 25 to 0.39% *w*/*v*. Overnight bacterial cultures grown in Mueller-Hinton broth was adjusted to a 0.5 McFarland turbidity standard (~1.5 × 10^8^ CFU/mL). Ten μL Mueller-Hinton broth containing approximately 5 × 10^4^ CFUs were added to 190 μL of tested 2-fold sample dilutions.

Positive control wells containing Mueller-Hinton broth inoculated with bacteria tested the growth of the pathogen. Negative BB control wells contained BB dilutions in Mueller-Hinton broth without bacteria. Negative Mueller-Hinton control wells containing only Mueller-Hinton broth without bacteria were used to test any possible contamination.

The optical density (OD) was determined at 630 nm using an EL ×808 absorbance microplate reader (BioTek Instruments, Inc., Winooski, VT, USA) just prior to incubation (t = 0) and 24 h after incubation (t = 24) at 37 °C. The OD for each negative BB control replicate well at t = 24 was subtracted from the OD of the same replicate test well with bacteria at t = 24. The growth inhibition at each ΒΒ dilution was determined using the formula % inhibition = [1 − (OD test well—OD of corresponding negative BB control well)] × 100. MIC was determined as the lowest bee bread concentration which results in 100% growth inhibition.

MIC values were expressed as mg/mL on dry weight basis. In order to measure the dry weight, the filtered aqueous phase of corresponding serial dilutions was dried for 24 h at 90 °C and then weighed. Drying was performed twice for each sample.

### 4.9. Determination of Minimum Bactericidal Concentration (MBC)

The MBC was determined by transferring a small quantity of sample contained in each replicate well of the microtiter plates to Mueller-Hinton agar plates by using a microplate replicator (BoekelScientific, Feasterville-Trevose, PA, USA). The plates were incubated at 37 °C for 24 h. The MBC was determined as the lowest bee bread concentration at which no grown colonies were observed [83].

### 4.10. Statistical Analysis

All results are expressed as the mean ± SD (*n* = 3). For statistical analysis, one-way analysis of variance (ANOVA) was applied, followed by Dunnett’s test for post-hoc analysis. All correlation analyses were conducted using Spearman’s correlation analysis. Values of *p* < 0.05 were considered to indicate statistically significant differences. All statistical analyses were performed using the SPSS version 13.0 statistical package (SPSS, Inc., Chicago, IL, USA).

### 4.11. Machine Learning Analysis for Prediction of Properties

A machine learning analysis was performed with the WEKA software in order to determine whether (i) antioxidant activity could be predicted from palynological composition, and (ii) antimicrobial activity could be predicted from palynological composition and/or antioxidant activity. Several linear regression and random forest models were assessed using five-fold cross-validation, after performing feature selection with a wrapper method (within WEKA). In order to further assess the robustness of our conclusions, the feature vectors of the 18 samples were shuffled and underwent feature selection and linear regression analysis to ensure that the high performance of certain models (based on actual data) could not be achieved by chance alone. Specifically, for the machine learning analysis and the sample clustering (see Section 4.12, below), the antimicrobial activity value of each sample was transformed to the dilution that the sample had to undergo, in order to achieve MIC or MBC. Samples with no MIC or MBC values were assigned zero values. A linear regression model was deemed to be predictive if (i) it achieved an R value above 0.5 for a certain combination of features, (ii) if the same combination of features achieved an R value above 0.5 for the random forest model as well, and (iii) if feature selection and linear regression modeling of the shuffled data could not achieve such high R values. Thus, very stringent criteria were applied.

### 4.12. Clustering of Samples Based on Their Properties

Clustering of the samples was performed with the pheatmap package in R. For the clustering of the 18 samples based on their palynological composition only, the Euclidean distance metric was applied.

## Figures and Tables

**Table 1:** TPC, TFC, DPPH, and ABTS^•+^ of bee bread samples.

Bee Bread Samples	TPC ^a^	TFC ^b^	DPPH ^c^	ABTS^•+ d^
1	9.56 ± 0.02.	3.88 ± 0.12	1.25 ± 0.04	0.51 ± 0.01
2	7.78 ± 0.13	3.78 ± 0.04	1.8 ± 0.07	1.80 ± 0.12
3	11.88 ± 0.06	5.49 ± 0.02	0.47 ± 0.03	0.55 ± 0.02
4	8.34 ± 0.13	5.02 ± 0.20	0.53 ± 0.03	0.53 ± 0.03
5	7.10 ± 0.08	3.61 ± 0.21	1.05 ± 0.09	1.50 ± 0.07
6	9.02 ± 0.08	4.60 ± 0.00	0.45 ± 0.03	0.50 ± 0.01
7	14.26 ± 0.31	4.82 ± 0.07	0.46 ± 0.08	0.60 ± 0.04
8	10.69 ± 0.08	4.62 ± 0.02	0.70 ± 0.09	0.80 ± 0.03
9	8.66 ± 0.26	3.63 ± 0.09	0.41 ± 0.04	0.45 ± 0.02
10	10.17 ± 0.01	2.56 ± 0.05	0.61 ± 0.02	0.50 ± 0.05
11	13.40 ± 0.43	5.27 ± 0.00	0.57 ± 0.05	0.51 ± 0.01
12	6.49 ± 0.04	3.54 ± 0.02	0.75 ± 0.05	0.81 ± 0.04
13	6.63 ± 0.05	3.31 ± 0.08	0.70 ± 0.05	1.02 ± 0.10
14	11.56 ± 0.03	4.75 ± 0.15	0.61 ± 0.08	0.62 ± 0.04
15	8.30 ± 0.13	2.34 ± 0.22	0.72 ± 0.03	0.72 ± 0.03
16	9.87 ± 0.07	3.18 ± 0.05	0.57 ± 0.05	0.63 ± 0.06
17	11.90 ± 0.03	3.92 ± 0.28	0.56 ± 0.09	0.62 ± 0.04
18	14.64 ± 0.26	4.18 ± 0.03	0.18 ± 0.02	0.38 ± 0.05

^a^ TPC is expressed as mg of gallic acid equivalents—GAEs/g sample; ^b^ TFC is expressed as mg of quercetin equivalent—QE/g sample; ^c^ IC_50_ values of BB samples in DPPH assay; ^d^ IC_50_ values of BB samples in ABTS^•+^ assay. All measurements were performed in triplicates and they are expressed as the mean values ± standard deviation (SD). Values are expressed on fresh weight basis.

**Table 2:** MIC and MBC values expressed in mg/mL.

Sample	*S. aureus*		*P. aeruginosa*		*S. typhimurium*		*K. pneumoniae*	
	MIC	MBC	MIC	MBC	MIC	MBC	MIC	MBC
1	22.6	45.2	45.2	45.2	90.4	>90.4	90.4	>90.4
2	22.6	45.2	22.6	22.6	22.6	45.2	11.3	22.6
3	48	48	24	24	12	24	24	24
4	9.9	19.8	19.8	19.8	9.9	9.9	9.9	9.9
5	23.5	23.5	23.5	23.5	23.5	23.5	23.5	23.5
6	45.6	45.6	45.6	45.6	45.6	91.2	45.6	>91.2
7	10.4	20.8	41.6	41.6	20.8	20.8	20.8	41.6
8	21.9	21.9	43.8	43.8	21.9	21.9	43.8	43.8
9	4.4	4.4	17.6	35.2	8.8	8.8	17.6	17.6
10	9.4	9.4	18.8	18.8	9.4	9.4	37.6	75.2
11	10.2	10.2	40.8	40.8	40.8	40.8	40.8	40.8
12	5	5	40	40	10	10	40	40
13	4.1	4.1	65.6	65.6	16.4	16.4	32.8	32.8
14	3.9	3.9	15.6	15.6	7.8	7.8	15.6	15.6
15	5.2	5.2	20.8	20.8	20.8	41.6	20.8	20.8
16	11.3	11.3	22.6	22.6	45.2	90.4	45.2	>90.4
17	23.3	23.3	46.6	46.6	46.6	93.2	46.6	>93.2
18	11	11	22	44	22	88	44	>88

MIC: minimum inhibitory concentration, MBC: minimum bactericidal concentration. Values are expressed on dry weight basis.

**Table 3:** Correlation coefficient (r) values estimated from correlation analysis between values of TPC, TFC, DPPH, and ABTS^•+^ of bee bread samples.

Methods	TPC	TFC	DPPH	ABTS^•+^
TPC		0.583 *	−0.586 *	−0.512 *
TFC			−0.444	−0.249
DPPH				0.719 **

* Correlation is statistically significant at *p* < 0.05; ** Correlation is statistically significant at *p* < 0.01.

**Table 4:** Correlation coefficient (r) values estimated from correlation analysis of TPC, TFC, DPPH, and ABTS^•+^ values with MIC and MBC values against four pathogens.

	*S. aureus*		*P. aeruginosa*		*S. typhymurium*		*K. pneumoniae*	
	MIC	MBC	MIC	MBC	MIC	MBC	MIC	MBC
TPC	0.211	0.149	0.196	0.348	0.180	−0.011	0.325	0.203
TFC	0.291	0.363	0.255	0.240	0.001	0.081	−0.112	0.084
DPPH	−0.033	0.013	0.057	−0.279	0.000	0.192	−0.224	0.049
ABTS^•^^+^	0.009	0.008	0.122	−0.249	0.156	0.229	−0.179	0.175

**Table 5:** Correlation coefficient (r) values estimated from correlation analysis of TPC, TFC, antioxidant activity, and antimicrobial activity values against dominant families of pollen content in each sample.

Pollen Family	TPC	TFC	DPPH	ABTS	*S. aureus*		*P. aeruginosa*		*S. typhimurium*		*K. pneumoniae*	
					MIC	MBC	MIC	MBC	MIC	MBC	MIC	MBC
*Boraginaceae*	0.500	0.500	0.000	−0.500	−0.500	−0.500	0.500	0.500	−0.500	-	−0.500	-
*Brassicaceae*	−0.300	−0.200	0.800	0.500	−0.900 *	−0.900 *	−0.100	−0.100	−0.100	−0.500	−0.100	0.500
*Cistaceae*	−0.600	−0.800	0.800	1.000 **	0.000	0.000	0.800	0.800	0.800	0.800	0.600	0.600
*Ericaceae*	0.500	−1.000 **	−1.000 **	−0.500	−0.500	−0.500	−1.000 **	−1.000 **	-	-	-	-
*Fabaceae*	−0.071	−0.321	0.000	−0.450	−0.536	−0.607	−0.893 **	−0.536	−0.857 *	−0.679	−0.393	−0.300
*Fagaceae*	1.000 **	0.000	−1.000 **	−0.949	0.400	0.200	0.200	0.400	0.400	0.400	0.400	-
*Guttiferae*	−0.300	−0.100	0.500	0.300	−0.200	−0.200	−0.300	−0.400	−0.300	−0.100	−0.100	−0.800
*Rosaceae*	−0.800	0.000	0.200	−1.000 **	0.200	0.400	0.200	0.200	−0.500	−0.500	−0.500	-

* Correlation is statistically significant at *p* < 0.05; ** correlation is statistically significant at *p* < 0.01.

## Data Availability

Not applicable.

## Supplementary Materials

The following are available online at https://www.mdpi.com/article/10.3390/antibiotics10050555/s1, Table S1: Palynological analysis of bee bread samples.
