# Supplementary material for: Biological Properties of Bee Bread Collected from Apiaries Located across Greece
Source: Antibiotics (Basel). 2021 May 10;10(5):555. doi: 10.3390/antibiotics10050555 (PMC8151309; doi:10.3390/antibiotics10050555)
Supplement: Supplementary file 1 [file antibiotics-10-00555-s001.zip › antibiotics-1217664-supplementary.pdf]

Supplementary Table 1. Harvest period, geographic location and palynologic analysis of bee bread samples. Numbers indicate % of pollen family

	Bee Bread Samples																	
	1	2	3	4	5	6	7	8	9	10	11	12	13	14	15	16	17	18
Harvest Period	Spring 2019	2018	Spring 2019	Spring 2019	Autumn 2019	Spring 2019	Spring 2019	Spring 2019	Spring 2019	Spring 2019	Spring 2019	Spring 2019	Spring 2019	Spring 2019	Summer 2019	Summer 2019	Summer 2019	Summer 2019
Geographic Location	Larissa	Thessaly	Magnesia	Larissa	Magnesia	Trikala	Heraklion	Kozani	Rethymno	Arta	Chania	Larissa	Larissa	Lasithi	Evoia	Arkadia	Chalkidiki	Mt.Athos
**Pollen Family**																		
Amaranthaceae					6.1				1		9.4					4.8		
Anacardiaceae												0.5						
Araliaceae		51.5			52.4				1	0.3	0.3					3		
Asteracea	0.5		0.8	0.5		0.4	24.5	0.8	4.1		5.7	0.2	0.6	0.2	1.5	6		
Begoniaceae				10.3			2.1	1.6	2.8			2		2.9				
Boraginaceae	3.1	1.6	3.9		8						54.8	0.5			34.5	16.2		
Brassicaceae			10.8	28.7	0.1	14.8	1	67.2	2.6	1.1	0.8	0.9		0.1	1	5	20.3	
Campanulacea							0.3					1.4						
Cistaceae									19.2		3.7	32.6	78.5	23.3		1.5		
Cupressaceae		24.3			17.5													
Ericaceae	17.7		2.6	2.8						41.6		23.8	1.2			1.4		
Fabaceae	1.1	2.9	18.5	2.7	11.5	10.8	11.3	3.8	30.4	27.9	6.4	4.5	4.4	6.6	2.2	16.5		
Fagaceae	4.3		1.9				24.2	4.9		1.5	1.7			13.7		2.1	14.1	99.8
Guttiferae	3.4	1.2	21.8	6.3		22.4	12.1	4.9	18.5	8.7	2.9	3.4		23.5	4.6	3.6		
Hydrophyllaceae			2.6	0.6										0.2				
Lamiaceae	3.7	0.1		4.6			0.6			1.1		3.6			10.6		19.9	
Liliaceae	2.3								4			14.1	13.5	5.5	3.5			
Myrtaceae		11.6												1.2				
Oleaceae						1.8	0.6			1.6	4.3	0.3			0.8		0.1	
Rhamnaceae												0.2						
Rosaceae	42.2	1.5	4.1	20.4	0.2	8	4.2	11	6.5	1.5	1.8	0.8				14.1	3.7	
Rutaceae										0.3	0.2	0.6						
Saliaceae	0.7		14.4	11.6		32.2	6.3	1.6		5		4.7		4.8	8.2	7.4	10.4	
Scrophulariaceae	8.8		5.4	2.8		5.9			7.7	5.8	3	3	0.9	16.6	2	5.9	14.5	
Umbelliferae/Apiaceae	3.8	2	8.1				12.1				3						3.7	0.1
Urticaeae												0.2						
Verbenacea							0.7					0.6			28		6	
Unknown	8.3	3.5	5.1	8.6	4.2	3.8		4.1	2.2	3.1	2.2	2.2	0.6	1.4	1.7	7.8	2.5	0.1
Zygophylaceae													0.3			4.9	4.8	
